# A strategy to improve arithmetical performance in four day-old domestic chicks (*Gallus gallus*)

**DOI:** 10.1038/s41598-017-13677-6

**Published:** 2017-10-24

**Authors:** Rosa Rugani, Maria Loconsole, Lucia Regolin

**Affiliations:** Department of General Psychology, University of Padua, Padua, Italy

## Abstract

A large body of literature shows that non-human animals master numerical discriminations, but a limit has been reported in a variety of species in the comparison 3*vs*.4. Little is known regarding the possibility of using “cognitive strategies” to enable this discrimination. The aims of this study were to investigate: whether domestic chicks discriminated 3*vs*.4, and if changes in stimuli presentation could improve chicks’ numerical performance. Newly hatched chicks were reared with seven identical objects. On day 4, they underwent 20 consecutive testing trials to assess their capability to discriminate 3*vs*.4. The objects were presented, one-by-one, to the chicks and hidden behind one of two identical panels. As expected, the chicks did not discriminate (Experiment 1). When objects were presented and hidden in groups comprising one or two objects (2 + 1)*vs*.(2 + 2), the chicks succeeded (Experiment 2). The grouping strategy did not help in the case of a harder discrimination of (3 + 1)*vs*.(3 + 2) (Experiment 3), unless chicks were allowed to rest for two hours between testing sessions (Experiment 4). Our results suggest that in some cases, the limits reported for numerical performance in animals do not depend on cognitive limitations but on attentional or motivational factors, which can be overcome employing simple procedural adjustments.

## Introduction

Numerical evaluation is one of the core abilities of the animal mind^1,2^. From the very first days of life, human and non-human animals can discriminate between groups on the basis of numerosity^3–7^. Such non-verbal numerical skills are based on two cognitive systems: one that represents small numerosities (the Object File System, *OFS*) and one that can also represent large numerical magnitudes (the Analogue Magnitude System, *AMS*). The *OFS* is an object-based attentional mechanism that precisely tracks individual objects by representing each of them as a distinct file in the working memory^8–11^. Spatio-temporal information^12–14^ and property/kind changes^13,15–20^ are used by the *OFS* to individuate and discern different objects.

Hence, such a system is not specific to number representation, though numbers are implicitly represented. This system’s signature is a set-size limit on the number (usually 3–4 for each set) of object-files that can be simultaneously attended to and held in the working memory^21^. Differences in the upper limit, 3 in the case of children and chicks^22,23^ and 4 in the case of adult monkeys^24,25^, have been attributed to maturational factors^1^.

The *AMS* can compute larger numerosities; its signature feature is to be ratio-dependent according to Weber’s law: as the ratio between the numbers to be discriminated becomes smaller, response times increase and accuracy decreases; e.g., to discriminate 1/2 would be easier than to discriminate 2/3^26^. The minimum discernible ratio diminishes over development in humans from 1/3 for newborns to 1/2 at six months, 2/3 for nine months, and 3/4 for preschool children^5,6,27^.

In non-verbal numerical cognition, 3*vs*.4 is a critical comparison. Some species succeed in this discrimination, such as: monkeys (*Macaca mulatta*), which discriminate 1*vs*.2; 2*vs*.3; 3*vs*.4, 3*vs*.5 but not 4*vs*.5; 3*vs*.6)^28^; mosquito fish (*Gambusia hoolbrooki*)^29^; domestic dogs (*Canis lupus familiaris*)^30^; Asian elephants (*Elephas maximus*)^31^; North Island robins (*Petroica longipes*)^32^; and orangutans (*Pongo pygmaeus*)^33^. However, a failure in discriminating 3*vs*.4 has been reported in: infants^34^; salamanders (*Plethodon cinereus*)^35^; and frogs (*Bombina orientalis*)^36^. Lack of discrimination between 3*vs*.4 could be explained by a difficult (too small) ratio to be discriminated by the *AMS* or by a too large absolute number of elements in each set to be computed by the *OFS*.

In previous studies, newly hatched domestic chicks, reared with identical objects, when presented with sets of 2*vs*.3; 1*vs*.4; 1*vs*.5; 2*vs*.4 objects that disappeared one-by-one behind separate panels, spontaneously inspected the panel occluding the larger set^37–39^.

Here, we used the same paradigm to assess chicks’ discrimination of 3*vs*.4, which for this species is reported as the upper limit in discriminating two consecutive numbers. Once this was determined, we changed the modality of stimuli presentation and used the same numerical comparison, in order to investigate whether grouping of the objects could improve their performance.

**Figure 1:**
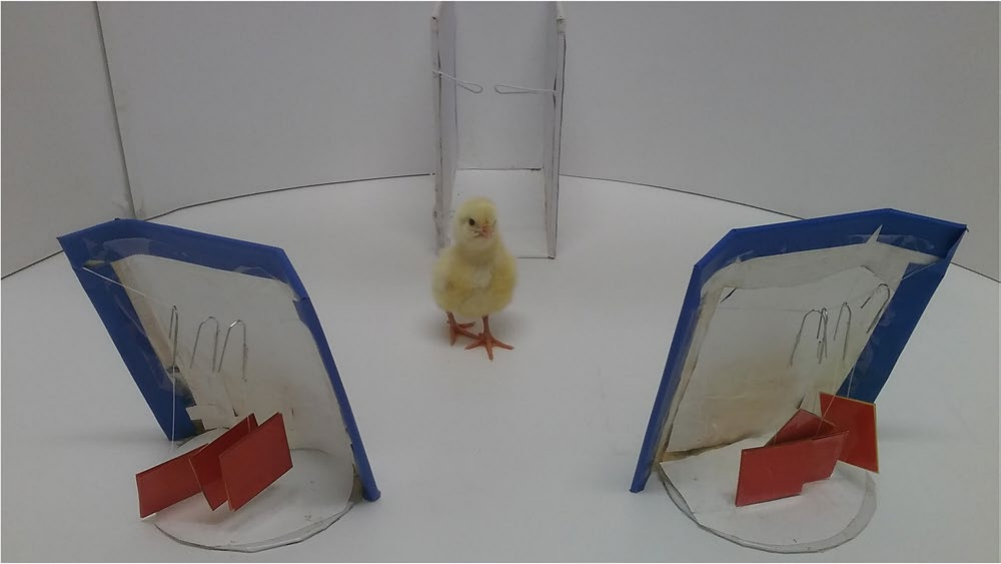
Apparatus used during training and test in all experiments. After the stimuli disappeared behind one of the two panels, the chick was left free in the arena. Each trial ended when the chick circumnavigate either panel.

We carried out four experiments. In Experiment 1, we asked whether four-day-old chicks could discriminate between three and four objects (i.e., 1 + 1 + 1*vs*.1 + 1 + 1 + 1). We reared newly hatched chicks with a group of seven identical objects. On day 4, the chicks underwent a free-choice test, which was composed of 20 consecutive trials. In each trial, we presented seven objects, identical to the rearing ones, one at a time; 3 of them disappeared behind a panel and the other 4 behind another panel (see Fig. 1). The chicks failed in this discrimination. This finding is consistent with previous results on non-verbal subjects that have shown a representational limit of three when required to track multiple objects through occlusion^34,40,41^. Furthermore, because previous results showed that infants using grouping increase their numerical abilities (e.g., infants can represent two spatially separated groups of two but not a single group of four; see the Discussion section), we asked whether grouping could also improve performance in birds. Therefore, in Experiment 2, objects (seven overall, as in Experiment 1) were presented and hidden as chunked into (2 + 1)*vs*.(2 + 2) units. In Experiment 3, we wanted to understand whether chicks could also master comparisons involving larger numerosities when objects are chunked. To this aim, we increased the number of objects by one in each unit (3 + 1*vs*.3 + 2). We hypothesized that a decrease in motivation/attention could affect the chicks’ performance. To investigate this aspect, in Experiment 4, we tested another group of chicks in the (3 + 1*vs*.3 + 2) discrimination, but the 20 testing trials were divided in four blocks, interspersed with two-hour breaks, with each block composed of five trials.

## Results

**Figure 2:**
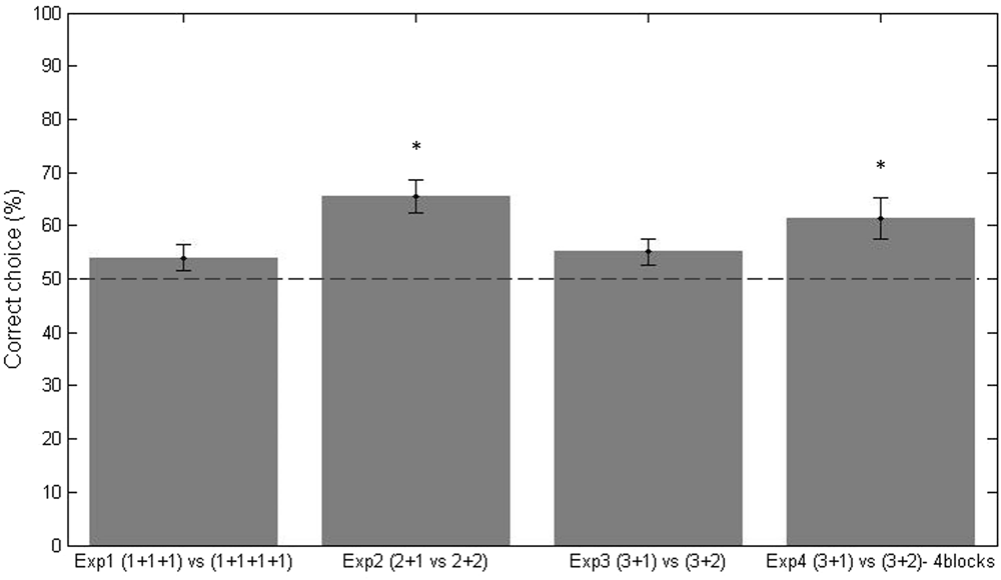
Results the four experiments. Mean percentage (and relative ES) of correct choices for each experiment. For experiment four the data of the four blocks have been merged. The dotted line represents chance level (50%) and * indicates p < 0.05 for the one-sample t-test.

On each test trial, we scored the first panel circumnavigated by the chicks. The number of trials in which each chick chose the panel hiding the larger set of objects (regarded as the correct choice) was calculated, and the percentages were computed as: number of correct choices/20 × 100. The mean (±SE) of the experimental group was compared with the chance level (50%) using one-sample t-tests. The results of all experiments are reported in Fig. 2.

In Experiment 1, in the sequential presentation of two sets of objects (1 + 1 + 1*vs*.1 + 1 + 1 + 1), the chicks did not preferentially circumnavigate the panel hiding the larger set: n = 10; M = 54.00, SE = 2.56; t(9) = 1.56; p = 0.15; d = 0.49. To ascertain any learning effect throughout the testing, the analysis was limited to the first five and the last five trials^37,42,43^. The birds’ performance in the first five trials (M = 62.00, SE = 7.57; t(9) = 1.59; p = 0.15; d = 0.5) and in the last five trials (M = 38.00, SE = 9.17; t(9) = −1.31; p = 0.22; d = −0.41) did not differ from chance. Overall, chicks are not able to discriminate between 3*vs*.4 objects when these objects are presented one by one.

In Experiment 2, which featured the grouped presentation of two sets of objects (2 + 1*vs*.2 + 2), the subjects (n = 11) circumnavigated the panel hiding the larger set of objects: M = 65.00, SE = 3.02; t(10) = 4.98; p < 0.001; d = 1.5. In order to assess whether the overall performance depended on learning, which might have occurred during testing, we considered the first five trials and the last five trials^37,42,43^. From the first trials, the chicks’ performance was statistically above chance (M = 67.27, SE = 4.88; t(10) = 3.54, p = 0.005; d = 1.07), and remained above chance in the last five trials (M = 69.09, SE = 7.32; t(10) = 2.61; p < 0.026; d = 0.79), as well.

Moreover, since in Experiments 1 and 2, chicks were required to discriminate between a set of three and a set of four elements, we compared the overall mean of correct responses by the two groups of chicks in these experiments. The t-test revealed that the chicks’ performance in the two experiments was statistically different t(19) = 2.75, p = 0.013; d = −1.2). This suggests that presentation modality can significantly improve the performance and confirms that a mechanism, such as grouping^44^, can improve numerical performance in an animal model.

In Experiment 3, featuring the grouped presentation of two sets of objects (3 + 1*vs*.3 + 2), the chicks (n = 11) did not circumnavigate the panel hiding the larger set of objects: M = 55.05, SE = 2.43; t(10) = 2.06; p = 0.067; d = 0.62. The birds’ performance differed from chance in the first five trials: M = 63.18, SE = 3.46; t(10) = 3.81; p = 0.003; d = 1.15, indicating that the chicks were at first able to discriminate between 3*vs*.5 objects. However, when we analyzed the last five trials, the chicks’ performance was at chance M = 58.18, SE = 5.69; t(10) = 1.43; p = 0.18; d = 0.43; this suggests that a decrease in motivation and/or attention may have influenced the performance.

In Experiment 4, which featured the grouped and time-separated presentation of two sets of objects (3 + 1*vs*.3 + 2), when the overall test trials were divided into four blocks of five trials each, a repeated measures ANOVA did not reveal any significant difference between blocks F(3,30) = 0.28; p = 0.84; *η* ^2^_*p*_ = 0.2 (First Block: M = 61.82%, SE = 6.30; Second Block: M = 58.18%, SE = 5.70; Third Block: M = 61.82%, SE = 5.01; Fourth Block: M = 65.46%, SE = 6.09). Data were merged and overall, the chicks circumnavigated the panel hiding the larger set (M = 61.36%, SE = 3.31; t(10) = 3.43; p = 0.01; d = 1.04).

To compare the effect of the test conditions in Experiments 3 and 4, in which the chicks were required to discriminate between a set of four and a set of five objects, we carried out a one-sample t-test. This revealed that the two experiments were not statistically different t(20) = −1.54, p = 0.14; d = −0.66 this is probably due to the fact that performance was above chance in the first trials for both experiments. Nevertheless, the overall performance differed from chance in Experiment 4, while it did not in Experiment 3. This difference may suggest that the procedure employed in Experiment 4 stabilized the performance throughout testing.

## Discussion

We assessed chicks’ discrimination of 3*vs*.4 and then we tested whether grouping could improve birds’ numerical skills. The results of Experiment 1 showed, as expected from previous studies, that the chicks failed in discriminating 3*vs*.4 objects that they had previously seen to disappear, one at a time, in two different locations. Experiment 1 confirms the limit of 3*vs*.4 in discriminating two consecutive numbers (similar results in discriminating two sets of visible objects has been reported in a previous study^23^). Lack of discrimination in 3*vs*.4 comparisons could be explained by a failure in discerning operated by either the Object File System (OFS) or the Analogue Magnitude System (AMS) (see the Introduction).

It has indeed been suggested that the modality of stimulus presentation can trigger activation of one or the other numerical systems^45^. On the basis of this hypothesis, when attention is focused on the distinct identity of the elements, by presenting one element at a time, the OFS would be activated and would account for numerical estimation. Whenever attention is directed towards the whole collection, presenting all the elements at the same time, the AMS would be activated and would support numerical discrimination^45^. According to this, the rearing experience of chicks with a whole group of objects could have elicited subsequent processing of those elements by AMS, even if they were presented one by one during the testing session^39^. Nevertheless, on the basis of Experiment 1, it was difficult to disentangle which system was elaborating the information.

However, data of the Experiment 2 suggest that discrimination in these tasks could be supported by the OFS. The signature limit of this system is indeed related to the span of the working memory. Here, exploiting a cognitive strategy (e.g., grouping) that has been shown to overcome the working memory span constraints in humans^46^, we showed that also chicks’ performance improved. In a grouped representation, individual objects are gathered together, but are still recoverable as individuals—this allows for the storage of more information in memory^47–52^. This strategy has been proved to implement numerical abilities in infants (fourteen-month-old infants^53,54^, thirteen-month-old infants^55^, and seven-month-old infants^56^). These studies indeed demonstrated that chunking allows retention of not only arrays of two and three hidden objects but also arrays of five, thereby overcoming the typically observed limits on working memory for un-chunked objects^40,41,57,58^. Nevertheless, whether or not grouping can implement numerical discrimination has never been investigated in a non-human species. The aim of Experiment 2 was to investigate the prerequisites for using a grouping strategy in an animal model. We presented chicks with the same numerical comparison used in Experiment 1 (3*vs*.4), but we changed the stimuli presentation by grouping objects. To this aim, each set was divided into two subsets: the set of three objects was split into a subset composed of a single object and into one composed of two objects; the set of four was divided into two identical sets each comprising two objects. In this case (3 + 1*vs*.2 + 2), the birds succeeded. Because of the modality of objects presentation, in which an object or a sub-groups of objects were presented one at a time, the system in charge of processing is probably the OFS. Indeed, although working memory is limited in adults and children, both have been shown to overcome these constraints through the use of chunking^46,59,60^. Furthermore, recent works have shown that chunking has its origins early in development. Fourteen-month-old infants successfully remembered the presence of four hidden objects when the objects were presented in two spatially grouped sets of two before they were hidden, but did not remember when these same objects were first presented in a single set of four^6,54^. Like adults, 14-month-old infants can chunk using their knowledge of object categories: they remembered four total objects when an array contained two tokens of two different types (e.g., two cats and two cars), but did not remember when the array contained four tokens of a single type (e.g., four different cats^6^).

Nevertheless, in our experiments, the same grouping strategy did not allow the discrimination of numerically larger sets (3 + 1*vs*.3 + 2; Experiment 3). Whenever the task became increasingly difficult, animals’ performance suffered from a decrease in motivation and/or attention. Moreover, failure in this test seems to suggest that chicks do not circumnavigate the panel that hides the two plural sets of objects, avoiding the one that hides a singular set. This supports the idea that, in selecting the panel hiding the larger group in Experiments 1 and 2, the birds used a computation based on numerousness and not on a discrimination between singular *vs*. plural. Interestingly enough, when the chicks’ performance was limited to the first five trials, this was significantly above chance, while it was at chance in the last five trials. This suggests that a decrease in motivation or attention may affect performance.

To directly assess the effect of timing, in Experiment 4, we tested a new group of birds in the same numerical task, but we changed the timing of the testing trials: the twenty testing trials were divided into four blocks of five testing trials each. In this case, the chicks were able to discriminate, and their performance remained stable above chance over blocks.

Overall, these data suggest that, in numerical tasks, the use of strategies such as grouping and evaluation timing may help in improving numerical competence. Nevertheless, it must be noted that, in this case, the use of a passive strategy (the birds were presented to grouped objects and did not group them by themselves) was successfully employed. Futures studies are needed in order to understand whether chicks, like human infants, can use any cues, such as color, size, or temporal regularities^61^, to spontaneously and actively chunk objects.

A deeper comprehension of the non-verbal numerical systems would not only add to our knowledge but would open a new and challenging educational issue. Indeed, it is well established that non-verbal numerical abilities (i.e., those calculations that can be solved in the absence of words expressing numbers) are available soon after birth and constitute the evolutionary foundations of more complex numerical reasoning^62,63^. From this viewpoint, non-verbal numerical competences are considered to be a building block for later mathematical abilities^62,63^. Educational interventions aimed at improving non-verbal numerical reasoning before children learn to count could constitute a valuable further intervention tool on infants with potential problems in mathematical comprehension.

## Methods

### Subjects

Forty-three female domestic chicks (*Gallus gallus*) took part in this study. They were obtained weekly, every Monday morning, from a local commercial hatchery (Agricola Berica, Montegalda, Vicenza, Italy) when they were only a few hours old. On arrival, chicks were housed in standard metal home cages (28 × 32 × 40 cm) at controlled temperature (28°–31 °C) and humidity (68%). Food and water were available *ad libitum*. Cages were constantly (24 h/day) illuminated by fluorescent lamps (36 W), located 45 cm above the floor. Each chick was placed ina cage together with an imprinting stimulus composed of seven (in Experiment 1 and 2) or nine (in Experiment 3 and 4) identical objects. These consisted of two-dimensional, about 1mm thick, red plastic squares (2.5 × 2.5 cm). Each object was suspended by a thin thread, at about 4–5 cm from the floor, so that it was positioned at about chicks’ head height, and therefore well visible. The objects were about 2 cm from one another. Objects identical to the ones used during imprinting were employed during training and testing. Previous studies demonstrated that, in this strain of chicks, this type of objects are very effective in producing social attachment through filial imprinting^4,15,16,64,65^. On Wednesday morning, two hours before training food was removed from the cages, while water was left available. At the end of training chicks were placed back in their home cages and, two hours later, they underwent testing individually.

### Apparatus

The experimental apparatus consisted in a circular arena (95 cm diameter, 30 cm outer wall height) whose floor was uniformly lined with white plastic sheets. Adjacent to the outer wall of the arena, there was a starting box (10 × 20 × 20 cm) used to shortly confine each chick before the trial started. The ceiling of the starting box was open, to allow the positioning of the chick at the beginning of each trial. The side walls of the box were fixed and covered with opaque plastic sheets. The side of the starting box facing the center of the arena consisted of a removable transparent glass partition (20 × 10 cm), so that while the chicks were kept there, they could see the inner arena and the stimuli. In front of the starting box, there was one (during training and re-training) or two (during testing) blue opaque vertical cardboard panel (16 cm × 8 cm). During training and re-training a single panel was positioned in the center of the arena, in front of and 35 cm away from the staring box. During testing, the two panels were positioned symmetrically, with respect to the front of the starting box, 35 cm away from it and 20 cm apart from one another. The panels were provided with 3 cm bent back edges on the two vertical sides, in order to prevent the chick from seeing the hidden objects before having circumnavigated the panel almost completely. In the experimental room temperature and humidity were maintained constant, respectively at 25 °C and 70%. The room was kept dark, except for the light coming from a 40 W lamp placed approximately 80 cm above the center of the experimental apparatus.

### Training

A preliminary training session took place on day three. Each chick, together with a single object, was placed in front of a single panel (about 20 cm away from it) in the apparatus. The object was held at the height of the chick’s head via a fine thread and kept visible to the chick. During this period, that lasted about five minutes, the bird was left free to move around and to get acquainted with the novel environment. Subsequently, the experimenter started to slowly move the object in the direction of the panel and, finally, behind it, until it completely disappeared from the chick’s sight. This sequence was repeated a few times, until the chick responded by following the attractor, for three consecutive times. Thereafter the chick was then confined in the starting box, and the transparent partition was put in place. As soon as the attractor had completely disappeared from chick’s sight, the transparent partition was lifted and the chick was set free to move wherever it wanted inside the arena. Every time it completely circumnavigated the panel, it was allowed to spend a few seconds together with the object. The sequence was then begun again and it was repeated until the chick promptly circumnavigated the panel for three consecutive times. The training session was identical for all the chicks and lasted about 30 minutes.

About one hour after the end of training each chick underwent test. This training procedure has been previously applied in researches in which the rearing objects have been used as attractors to test cognitive abilities in day-old chicks^4,15,16,37–39,64,65^.

### Test

A complete testing session comprised 20 trials for all experiments. In Experiments 1, 2 and 3 chicks underwent 20 consecutive trials; in Experiment 4 the 20 trials were divided in 4 block of 5 trials each. At the beginning of each trial, the chick was confined to the starting box with the transparent partition in place,. The chick was shown two sets of objects: one made of three and the other made of four objects. Depending on the experiment, the chick was either presented with an object or with a sub-group of objects at a time. Every object or sub-group of the first set was placed about 10 cm from the front of the holding box and then made to disappear behind either panel. Immediately after it disappeared the next object or sub-group was introduced into the arena. In this way, all the objects of the first set were made to disappear behind the same panel. Then, the identical procedure was repeated for the second set that was made to disappear behind the other panel. In this way both sets disappeared, each behind a different panel. In order to avoid the possibility that chicks relied only on the first or on the last chunk, ignoring the other one, we randomized which set was hidden as first (either the larger or the smaller) and the panel (left or right one) behind which the first set was hidden. Moreover no set was ever hidden twice consecutively behind the same panel^15,16,37–39^. In Experiment 4, randomization was maintained and the sequence was divided in 4 blocks. Each object or sub-group was kept in front of the starting box for 3 s and then it took 3 s for it to be moved back behind the panel (6 s overall). About 2 s elapsed from the disappearance of one object or grouped objects and the appearance of the next one. About 3 s after the disappearance of both sets, the transparent partition was lifted from above and the bird was left free within the arena.

A choice was defined as when at least the head and ¾ of the chick’s body had entered the area behind either panel (beyond the side edges); at which point the trial was considered over. Only the first panel visited was scored. At the end of each trial, chicks were allowed to spend 1–2 s with their ‘social companions’ behind the panel chosen by the chick. Thereafter the chick was positioned back in the starting box with both the transparent partition in place. After a few second the subsequent trial began. This procedure was carried out such that each chick underwent a complete testing session of 20 valid trials. If the chick did not approach either panel within three min, the trial was considered null and discarded and it was repeated immediately afterwards. After three consecutive null trials, the chick was placed back within its own rearing cage (in the presence of the imprinting objects) at least for one hour before being resubmitted to further testing trials. Whenever the same bird made to register another three consecutive null trials, it was discarded from the experiment. This happened for 12% of chicks.

During testing chicks’ behavior was observed and scored by a monitor connected to a video camera so as not to disturb the chicks by direct observation. The trials were entirely video-recorded so that a second, blind experimenter could score the chicks’ performance off-line^15,16,37–39^. On-line and off-line scoring was found to be highly consistent with one other.

## Ethic Statement

The experiments complied with all applicable national and European laws concerning the use of animals in research and were approved by the Italian Ministry of Health (permit number: 32662 emitted on 10/1/2012). All procedures employed in the experiments included in this study were examined and approved by the Ethical Committee of the University of Padua (Comitato Etico di Ateneo per la Sperimentazione Animale –C.E.A.S.A.) as well as by the Italian National Institute of Health (N.I.H).

### Data availability statement

The datasets generated during and/or analyzed during the current study are available from the corresponding author on reasonable request.
